# Evaluation of three prediction formulas of 24-hour urinary sodium excretion in Chinese residents: a systematic review and meta-analysis

**DOI:** 10.1017/S1368980024000168

**Published:** 2024-02-02

**Authors:** Zijing Qi, Shuai Tang, Beike Wu, Yanxing Li, Hongmei Yang, Kunbo Wang, Zhifang Li

**Affiliations:** 1 School of Public Health, Shanxi Medical University, Taiyuan 031000, China; 2 School of Public Health, Harbin Medical University, Harbin 150081, China; 3 Department of Public Health and Preventive Medicine, Changzhi Medical College, Changzhi 046000, China; 4 CLASS 2202, Xiangya School of Medicine, Central South University, Changsha 410000, China

**Keywords:** Meta-analysis, Sodium, Spot urine, 24-h urine, Chinese residents

## Abstract

**Objective:**

To determine the appropriateness of three widely used formulas estimating 24-h urinary Na (24hUNa) from spot urine samples in the Chinese population.

**Design:**

Systematic review and meta-analysis.

**Setting:**

Literature review was conducted to identify studies for estimating 24hUNa using the Kawasaki, Tanaka and INTERSALT formulas simultaneously in PubMed, Embase and the Cochrane library databases. The mean difference (MD) and correlation coefficients (r) between measures and estimates from different formulas were assessed.

**Participants:**

Information extraction and quality assessment were performed in thirteen studies involving 8369 subjects.

**Results:**

Two studies which affected the overall robustness were excluded in the ‘leave-one-out’ sensitivity analyses. Within the final meta-analysis included eleven studies and 7197 participants, 36·07 mmol/d (95 %CI 16·89, 55·25) of MD was observed in the Kawasaki formula, and –19·62 mmol/d (95 %CI –37·37, –1·87) in the Tanaka formula and –35·78 mmol/d (95 %CI –50·76, –20·80) in the INTERSALT formula; a pooled r-Fisher’s *Z* of 0·39 (95 %CI 0·32, 0·45) in the Kawasaki formula, 0·43 (95 %CI 0·37, 0·49) in the Tanaka formula and 0·36 (95 %CI 0·31, 0·42) in the INTERSALT formula. Subgroup analyses were conducted to explore the possible factors affecting the accuracy of the formula estimation from three mainly aspects: population types, Na intake levels and urine specimen types.

**Conclusions:**

The meta-analysis suggested that the Tanaka formula performed a more accurate estimate in Chinese population. Time of collecting spot urine specimens and Na intake level of the sample population might be the main factors affecting the accuracy of the formula estimation.

Globally, excessive salt intake is recognised as a public health issue^(1)^. In 2013, the WHO recommended for a 30 % reduction in daily salt intake, aiming for a population salt reduction target of less than 5 g per day per individual by 2025, as a global salt reduction initiative^(2)^. To achieve this goal, a series of salt reduction strategies have been promoted in China^(3)^. Regularly monitoring population salt (Na) intake^(4)^ is a common approach to assess the effectiveness of salt reduction strategies^(5)^. While 24-h urine collection is esteemed as the gold standard for assessing population Na intake, its practicality in extensive epidemiological research^(6)^ is curtailed by inherent challenges such as the significant burden of collection, elevated costs and the prevalence of incomplete collections^(6)^. The measurement of Na in a spot urine has the potential to estimate the 24-h urinary Na (24hUNa)^(7)^, when assessing Na intake at the population level^(8)^. Currently, there are three widely used formulas estimating 24hUNa from spot urine samples among Chinese population including the Kawasaki formula^(9)^, the Tanaka formula^(10)^ and the INTERSALT formula^(11)^. The appropriateness of the three formulas has not been systematically evaluated due to their diverse predictive results in different studies^(12–19)^. The aim of this meta-analysis is to identify which formula could produce a more accurate estimate in the Chinese population.

## Methods

### Databases and search terms

**Fig. 1:**
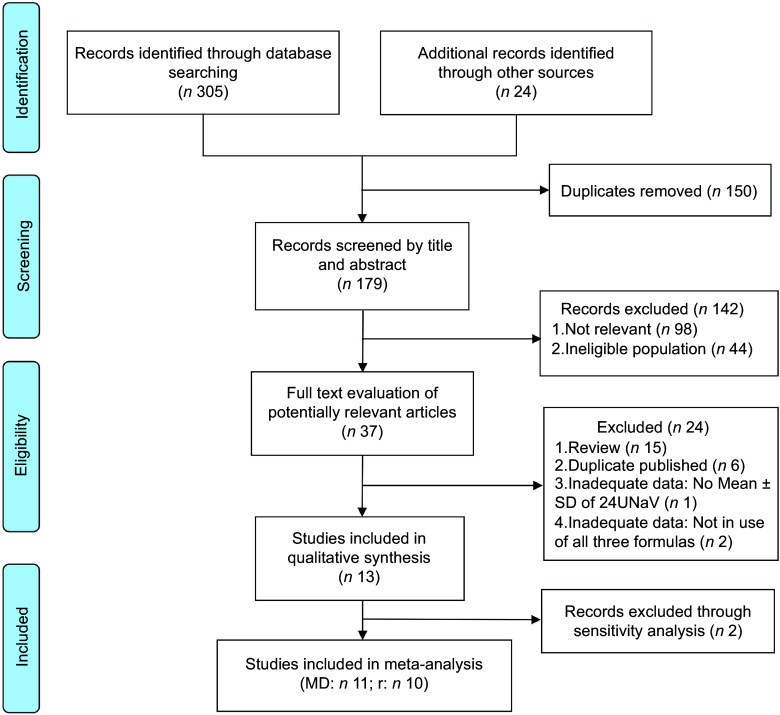
PRISMA flow diagram of the screening procedure followed to identify eligible studies. PRISMA, Preferred Reporting Items for Systematic Reviews and Meta-Analyses; MD, mean difference; r, correlation coefficient

The systematic review was guided by the Preferred Reporting Items for Systematic Reviews and Meta-Analyses checklist^(20)^. A combination of terms was used to search the titles and abstracts of publications in PubMed, Embase and the Cochrane library, including ‘spot urine’ AND ‘sodium’ AND ‘China’ (Chinese residents). Search results were imported to EndNote X9 (Clarivate Analytics, 2019) for screening and extraction. The literature screening process is illustrated in Fig. 1.

### Inclusion and exclusion criteria

All eligible studies need to meet the criteria below:

1. Type of participants. Participants included healthy or high-risk people of any age from any region of China; and
2. Type of outcome measures. Studies reported mean Na intake by estimation of spot urine and 24-h urine collection specimens at the same time; and
3. Method of validation. The Kawasaki, Tanaka and the INTERSALT formulas were used to report the estimates and measures (mean ± sd) at the same time; and
4. Language. All studies were published in English; and
5. Studies were eligible if published before November 2022.

Studies were excluded if they did not provide the measure of 24hUNa and estimates from spot urine using the three formulas simultaneously.

### Study screening and data extraction

All titles and abstracts of included studies were initially screened by two reviewers (ZQ, ST), and the full text of potentially relevant articles was further reviewed. Then, the two reviewers independently extracted relevant data as follows: the first author, publication year, area studied, study population, sample size, female proportion, mean age (age range), fasting or not, type of spot urine sample, measured 24hUNa (mean ± sd), the formula used, estimated 24hUNa (mean ± sd) and correlation coefficients (r). Reviewers resolved disagreements through consulting senior researchers (ZL) if necessary, during the study screening, review and data extraction. Details of the included thirteen studies are summarised in Table 1.

**Table 1:** Characteristics of included studies

First author, year	Area studied	Population studied	Mean age (years)	Sample size	Female proportion	Measured 24hUNa (mmol/d)		Fasting	Urine type	Kawasaki			Tanaka			INTERSALT		
										24hUNa (mmol/d)		***r***	24hUNa (mmol/d)		***r***	24hUNa (mmol/d)		***r***
Xiaofu Du 2021^(15)^	Zhejiang	Healthy adults	46·7	1424	51·1 %	165·7	71·5	Yes	MU	183·8	56·7	0·3	142·9	35·6	0·3	133·6	33·7	0·3
Jianwei Xu 2020^(14)^	Zhejiang	Healthy adults	46·7	1428	50·9 %	167·2	74·7	Yes	MU	184·1	56·9	0·4	143·1	35·6	0·3	133·8	33·9	0·4
Jianwei Xu 2020^(27)^	Shandong	Healthy adults	43·8	1671	50·0 %	176·4	79·1	Yes	MU	235·0	70·7	0·4	173·8	41·1	0·4	152·0	36·5	0·4
Long Zhou 2017^(28)^	Jiangxi	Healthy adults	51·1	141	94·3 %	220·8	78·5	N/A	MU	246·1	66·8	0·3*	143·6	24·7	0·4*	183·7	39·0	0·2*
Ying Zhang 2018^(25)^	Beijing	Healthy adults	32·2	85	62·4 %	198·2	83·0	N/A	SMU, CU	231·6	67·7	0·3*	193·93	50·2	0·5*	136·5	29·9	0·4*
Jie Dong 2019^(16)^	Hunan	Healthy adolescents	12·1	284	47·9 %	124·9	42·5	Yes	MU	195·0	48·6	0·2	124·3	24·9	0·6	115·6	31·3	0·3
Xin Zhang 2021^(26)^	Sichuan	General Tibetan adults (hypertension 54·5 %)	51·2	323	60·9 %	198·8	38·7	Yes	SMU	204·2	47·5	0·4	158·0	29·6	0·4	141·7	33·3	0·3
Yaguang Peng 2016^(29)^	Shanxi	General adults (hypertension 56·9 %)	53·2	116	68·1 %	275·8	107·4	Yes	MU	243·6	64·0	0·2	175·6	33·6	0·3	154·2	38·0	0·2
Ni Qian 2021^(18)^	Dalian	Hypertensive patients	55·6	1154	46·0 %	129·1	54·8	N/A	MU	213·9	65·7	N/A	167·4	40·4	N/A	142·6	38·7	N/A
Yan Sun 2020^(19)^	8 provinces	Hypertensive patients	60·0	290	53·4 %	145·7	66·7	NO	CU	216·4	68·7	0·6	169·4	41·9	0·6	137·81	40·1	0·5
Beike Wu 2022^(30)^	Shanxi	Stroke patients	64·8	281	49·5 %	180·7	67·6	NO	CU	218·7	70·5	0·4	169·3	41·6	0·4	142·86	44·6	0·4
Wenxia Ma 2017^(13)^	Shaanxi	High-risk elder patients of stroke	67·5	365	57·5 %	162·0	70·4	NO	CU	193·9	70·7	0·4	378·7	109·9	0·4	130·0	41·5	0·3
Yi Zhao 2019^(17)^	Ningxia	Hypertensive patients	69·8	807	42·5 %	131·7	66·1	NO	CU	1906·5	726·5	N/A	927·0	270·0	N/A	128·7	49·1	N/A

24hUNa, 24-h urinary Na; MU, morning urine; SMU, second morning urine; CU, casual spot urine; *r*, Pearson correlation coefficient between measured and formula-based estimated 24hUNa excretion, except for Spearman correlation efficient with *; N/A, no statistic.

### Statistical analysis

Pooled mean difference (MD) and pooled r-Fisher’s *Z* of the three formulas were calculated using the Sidik–Jonkman method^(21)^ for random-effect models. Sensitivity analyses were carried out by the ‘leave-one-out’ method, which removed one study at a time to check the robustness of the result. If the pooled results showed relatively large biases when a study was excluded, it suggests that the study had a significant impact on the overall results of meta-analysis, leading to its exclusion. Heterogeneity between studies was reflected by *I* ^2^ tests. Subgroup analyses were also implemented to detect potential sources of heterogeneity: (1) population subgroup (healthy or high-risk population), (2) Na intake subgroup (more than 10 g/d group or no more than 10 g/d group) and (3) urine sample type subgroup (casual spot urine, morning urine or fasting morning urine). The meta-analysis was conducted at the population level using software RevMan 5.3 (Computer program, version 5.3. Copenhagen: The Nordic Cochrane Centre, The Cochrane Collaboration, 2014) and Prism 9.3.1 (GraphPad Software).

Na excretions were converted into mmol/d for consistency, using the following equations: 1 mmol Na = 23 mg Na and 1 mg Na = 2·54 mg salt^(1)^. Spearman correlation coefficients were converted to Pearson correlation coefficients for consistency using the following equations: *r* = 2×sin(r_s_ × *π*/6)^(22)^. Correlation coefficients (r) reported in each study were converted into Fisher’s Z using the following specific formula^(23)^. The conversion formula between the correlation coefficient and Fisher’s *Z* is as follows: *Z* = 0·5×[ln(1 + r)–ln(1–r)]. The variance of *Z* is: Vz = 1/ (*n*–3). The standard error of *Z* is: SEz = √ Vz.

### Quality assessment

Study quality was independently assessed by two reviewers (ZQ, ST) using a modified tool for evaluating dietary intake validation studies^(24)^. The tool rates the studies through five domains on a scale from 0 (poorest quality) to 7 (highest quality), with the following interpretations: excellent if the score ≥5·0; good if ≥3·5 and <5·0; acceptable or reasonable if ≥2·5 and <3·0; and poor if <2·5.

The quality assessment domain was modified to facilitate the estimated 24hUNa by spot urine^(1)^, through the consideration of variables as below: (1) sample, with a maximum of 1 point: 0·5 point when the sample size was of more than 100 individuals; and 0·5 point allocated when the sample was homogeneous for sex, age and population; otherwise, sample was considered non-homogeneous and given a score of 0; (2) statistics. A maximum of 3 points was allocated; 1 for MD between estimated and measured values; 0·5–1 point according to the correlation used (r, ICC); 0·5–1 according to the agreement used Bland–Altman plots or rate of misclassification. (3) Data collection with a maximum of 1 point: 0·5 point if verbal or written instructions were conducted to collect urine to the participants; plus 0·5 when spillage or missed voids were assessed post-collection. (4) Seasonality. Addition of 0·5 point only when considered in the validation design. (5) Supplements. Addition of 1·5 points when the validation study considered supplements. The results of quality assessment are shown in Table 2.

**Table 2:** Quality assessment of the included studies

Domain	Specific item	**Xiaofu Du 2021** ^(15)^	**Jianwei Xu 2020** ^(14)^	**Jianwei Xu 2020** ^(27)^	**Long Zhou 2017** ^(28)^	**Ying Zhang 2018** ^(25)^	**Jie Dong 2019** ^(16)^	**Xin Zhang 2021** ^(26)^	**Yaguang Peng 2016** ^(29)^	**NiQian 2021** ^(18)^	**Yan Sun 2020** ^(19)^	**Beike Wu2022** ^(30)^	**Wenxia Ma 2017** ^(13)^	**YiZhao 2019** ^(17)^
Sample	*N* > 100	0·5	0·5	0·5	0·5	0	0·5	0·5	0·5	0·5	0·5	0·5	0·5	0·5
	Non-homogenous sample (sex, age, population)	0·5	0·5	0·5	0	0·5	0	0·5	0·5	0	0	0	0	0
Statistics	Mean difference	1	1	1	1	1	1	1	1	1	1	1	1	1
	Correlation	1	1	1	0·5	0·5	1	1	1	0·5	0·5	1	1	0·5
	Agreement	1	0·5	1	1	1	1	1	0·5	1	1	1	0·5	0·5
Data collection	Verbal or written instructions to collect urine to the participants	0·5	0·5	0·5	0·5	0·5	0·5	0·5	0·5	0·5	0·5	0·5	0·5	0·5
	Spillage or missed voids assessed post-collection	0·5	0·5	0·5	0·5	0·5	0·5	0·5	0·5	0·5	0·5	0·5	0·5	0·5
Seasonality	Considered	0	0	0	0	0	0	0	0	0	0	0	0	0
Supplements	Included and data considered in analysis	0	0	0	0	0	0	0	0	0	0	0	0	0
Score^*^		5	4·5	5	4	4	4·5	5	4·5	4	4	4·5	4	3·5
Quality^†^		E	G	E	G	G	G	E	G	G	G	G	G	G

* Score interpretations: ≥5·0, excellent quality; ≥3·5 and < 5·0, good quality; ≥2·5 and < 3·5, acceptable quality; < 2·5, poor quality.

† E, excellent; G, good.

## Results

### Literature selection

One hundred seventy-nine articles were retrieved initially, of which 142 articles were excluded, leaving thirty-seven studies for full-text review. Finally, thirteen studies were included in this review, and eleven studies were conducted in the meta-analysis. The study selection process is presented in Fig. 1.

### Study characteristics

Through literature selection, thirteen studies involving 8369 participants from fifteen provinces of China (Beijing^(19,25)^, Shanghai^(19)^, Tianjin^(19)^, Sichuan^(19,26)^, Shandong^(19,27)^, Henan^(19)^, Xinjiang^(19)^, Gansu^(19)^, Zhejiang^(14,15)^, Jiangxi^(28)^, Hunan^(16)^, Shanxi^(29,30)^, Dalian^(18)^, Ningxia^(17)^, Shaanxi^(13)^) were initially included. Among all included studies, six (46·15 %) were conducted in the healthy population^(14–16,26–28)^, seven (53·85 %) included the high-risk population, five (38·46 %) were associated with the hypertensive patients^(12,17,18,26,29)^ and two (15·38 %) involved the stroke high-risk population^(13,30)^. Twelve studies (92·31 %) recruited adults, while one focused on adolescents^(16)^. The detailed characteristics of included thirteen studies are presented in Table 1.

### Quality assessment

All included studies were of good quality, with three studies rated as excellent and the others as good. The details are presented in Table 2.

### Sensitivity analyses

**Fig. 2:**
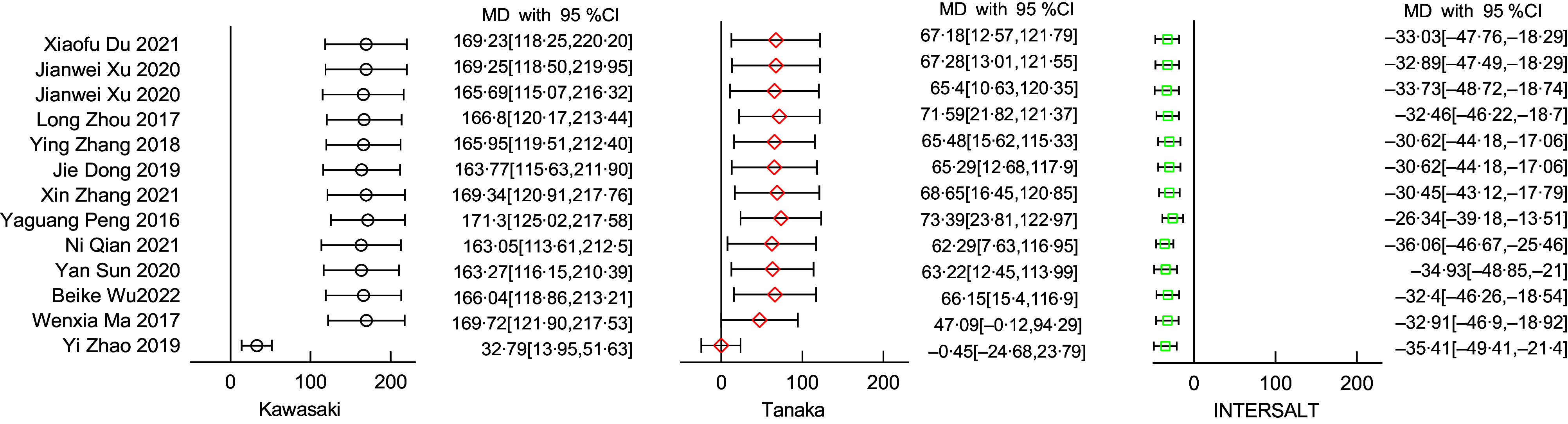
Sensitivity analyses in use of the ‘leave-one-out’ method: pooled estimates were from random-effects models with removing one study at a time

As shown in Fig. 2, sensitivity analysis using the ‘leave-one-out’ approach excluded Yi Zhao *et al*.’s study^(17)^and Wenxia Ma *et al*.’s study^(13)^ which had great impact on the robustness of the overall results. Table 3 shows the impact of the two excluded studies on the meta-analysis.

**Table 3:** Sensitivity analyses of mean difference (MD) in different formulas

Formula		Min of MD	Max of MD	Pooled MD
Kawasaki	Sensitivity 1	−2·10	1774·80	155·74
	*n* 13, *I* ^2^ = 100 %	–9·55, 5·35	1724·47, 1825·13	111·32, 200·15
	Sensitivity 2	−2·10	84·80	32·79
	*n* 12, *I* ^2^ = 99 %	–9·55, 5·35	79·86, 89·76	13·95, 51·63
	Sensitivity 3	5·40	84·80	36·07
	*n* 11, *I* ^2^ = 99 %	–1·28, 12·08	79·86, 89·76	16·89, 55·25
Tanaka	Sensitivity 1	−0·60	795·30	60·16
	*n* 13, *I* ^2^ = 100 %	–6·33, 5·13	776·12, 814·48	12·46, 107·86
	Sensitivity 2	−0·60	216·70	−0·45
	*n* 12, *I* ^2^ = 99 %	–6·33, 5·13	203·31, 230·09	–24·68, 23·79
	Sensitivity 3	−0·60	−100·20	−19·62
	*n* 11, *I* ^2^ = 99 %	–6·33, 5·13	–120·68, –79·72	–37·37, –1·87
INTERSALT	Sensitivity 1	−3·00	−121·60	−32·79
	*n* 13, *I* ^2^ = 100 %	–8·68, 2·68	–142·33, –100·87	–45·96, –19·61
	Sensitivity 2	−7·85	−121·60	−35·41
	*n* 12, *I* ^2^ = 98 %	–16·80, 1·10	–142·33, –100·87	–49·41, –21·40
	Sensitivity 3	−7·85	−121·60	−35·78
	* n* 11, *I* ^2^ = 99 %	–16·80, 1·10	–142·33, –100·87	–50·76, –20·80

Sensitivity 1, including all available thirteen studies; Sensitivity 2, excluded one study^(17)^ that had a large impact on the overall results from thirteen studies; Sensitivity 3, excluded two studies^(13,17)^ that had large impacts on the overall results from thirteen studies.

### Overall result

**Fig. 3:**
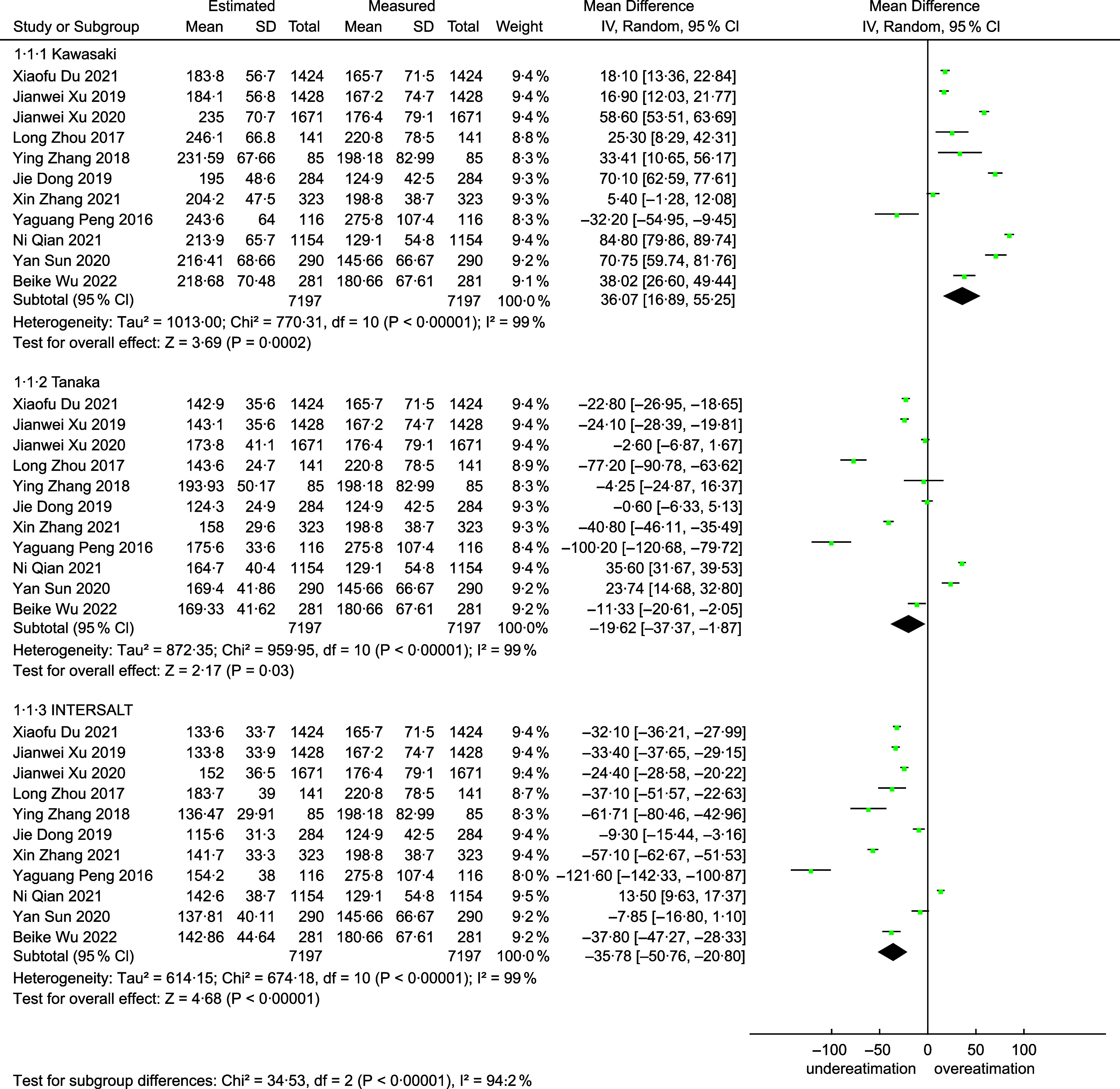
Forest plot of mean difference (MD) between measures and estimates from different formulas

**Fig. 4:**
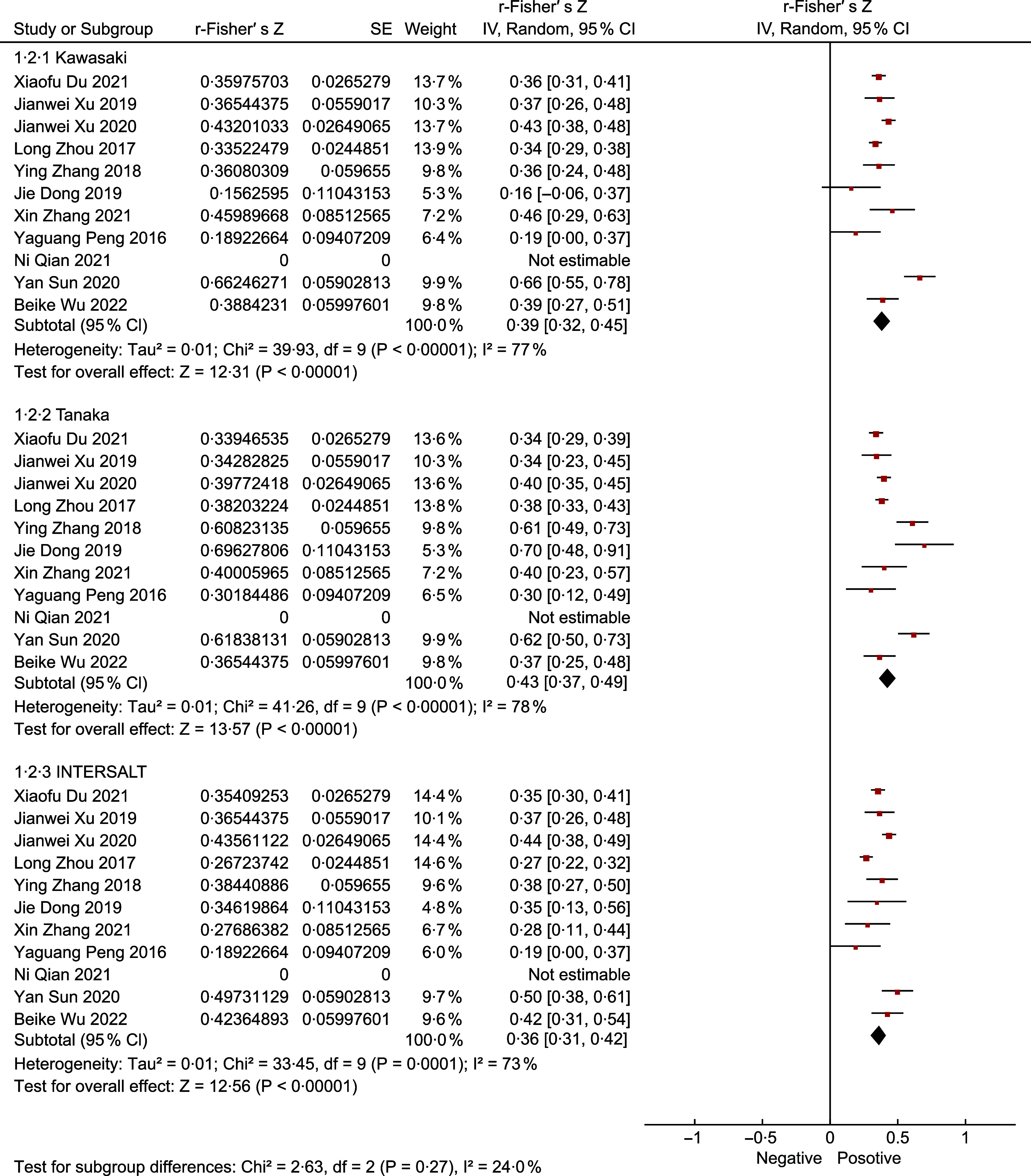
Forest plot of correlation (r) between measures and estimates from different formulas

The final meta-analysis as shown in Fig. 3, which included eleven studies and 7197 participants, observed a MD of 36·07 mmol/d (95 %CI: 16·89, 55·25) in the Kawasaki formula, –19·62 mmol/d (95 %CI –37·37, –1·87) in the Tanaka formula, and –35·78 mmol/d (95 %CI −50·76, –20·80) in the INTERSALT formula. Of the eleven included studies, ten (90·90 %) provided correlation coefficients between the estimates and the measures. The meta-analysis (Fig. 4) showed a pooled r-Fisher’s *Z* of 0·43 (95 %CI 0·37, 0·49) in the Tanaka formula, 0·39 (95 %CI 0·32, 0·45) in the Kawasaki formula and 0·36 (95 %CI 0·31, 0·42) in the INTERSALT formula. According to the interpretation of the correlation coefficients from Psychology^(31)^, the correlation coefficient converted from r-Fisher’s *Z* showed that the Tanaka formula (0·41) performed a moderate correlation, while the Kawasaki formula (0·37) and the INTERSALT formula (0·35) performed a weak correlation. Through the comparison of the statistical significance of the difference between two independent correlation coefficients, the Tanaka formula had a higher correlation than the Kawasaki formula (0·41 *v*. 0·37, *P* = 0·01) and the INTERSALT formula (0·41 *v*. 0·35, *P* < 0·01).

### Subgroup analysis

For those consuming over 10 g/d, the Kawasaki formula indicated a MD of 22·14 mmol/d (95 % CI –3·97, 48·25), while the Tanaka and INTERSALT formulas showed –38·73 mmol/d (95 % CI –63·33, –14·13) and –55·02 mmol/d (95 % CI –74·62, –35·41), respectively. In contrast, among participants with an intake of 10 g/d or less, the Kawasaki formula revealed a MD of 53·11 mmol/d (95 % CI 27·84, 78·37); for the same group, the Tanaka and INTERSALT formulas reported 2·30 mmol/d (95 % CI -24·06, 28·66) and –13·87 mmol/d (95 % CI –34·49, 6·76), respectively. Examining the casual spot urine group, MD of 54·42 mmol/d (95 % CI 22·35, 86·50) was observed for the Kawasaki formula, 6·22 mmol/d (95 % CI –28·15, 40·59) for the Tanaka formula and –34·81 mmol/d (95 % CI –62·67, –6·96) for the INTERSALT formula. Meanwhile, in the fasting morning urine group, the observed MD were 24·17 mmol/d (95 % CI 2·42, 45·93) using the Kawasaki formula, –28·96 mmol/d (95 % CI –43·57, 14·35) with the Tanaka formula and –42·96 mmol/d (95 % CI –57·25, –28·67) with the INTERSALT formula.

**Fig. 5:**
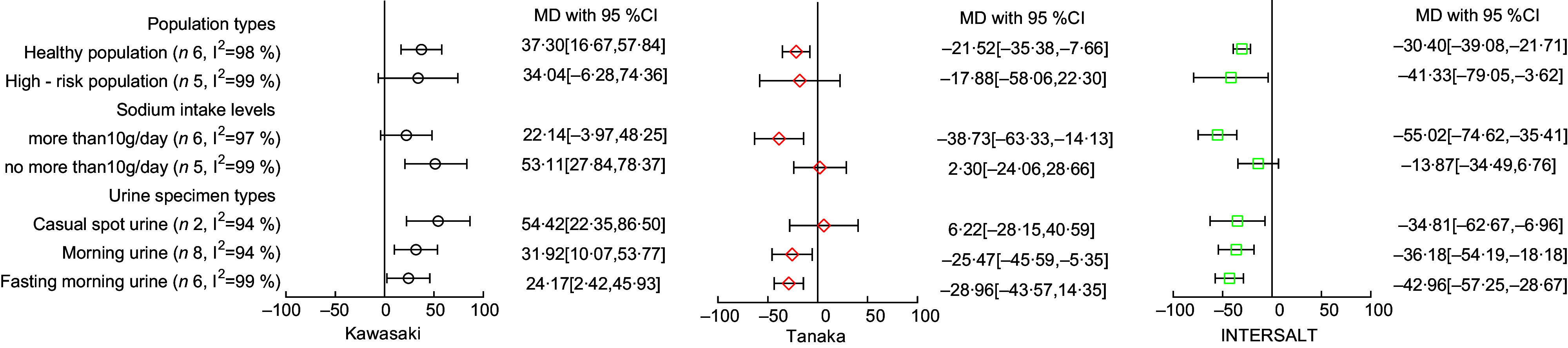
Forest plot of mean difference (MD) from different formulas in different subgroups

The detailed results are summarised in Fig. 5.

## Discussion

Using spot urine samples could be a convenient alternative to 24-h urine collections for monitoring Na intake at the population level^(26,32)^. The three formulas in the meta-analyses all showed moderate correlations (0·36–0·43) between the estimated and measured 24hUNa, slightly lower than those observed in the Italian population (0·62–0·70)^(33)^ and slightly higher than the Portuguese population (0·25–0·36)^(34)^. In a diverse population from eleven countries^(35)^, the Kawasaki formula was considered a reliable alternative for estimating 24hUNa while the INTERSALT formula was seen as an acceptable alternative for monitoring Na intake/excretion in the French population^(36)^. Several foreign validation studies^(37,38)^ also identified that the INTERSALT formula which derived from data across fifty-two centres in thirty-two countries perform better under complex ethnic compositions. This meta-analysis in the Chinese population suggested the Tanaka formula showed a less bias (–19·62 mmol/d) compared with the Kawasaki formula (36·07 mmol/d) and the INTERSALT formula (–35·78 mmol/d).

Subgroup analysis identified two key factors that might influence the accuracy of formula estimates. First, Na excretion exhibited a diurnal variation^(39)^, implying that the time of urine sample collection could impact the overestimation or underestimation of 24-hour urinary sodium excretion (24hUNa)^(40,41)^. The Kawasaki formula initially was established based on fasting second morning urine specimens^(9)^, which had a lower Na concentration^(27,42)^, while the Tanaka formula and the INTERSALT formula were established based on casual spot urine specimens which took the daily change of urine Na into account. Therefore, the Kawasaki formula produced higher estimates when collecting casual spot urine specimens, and the Tanaka and the INTERSALT formula produced lower estimates when fasting morning urine was collected. Second the level of Na intake appears to play a role in estimation accuracy. Numerous studies^(12,18,19,29,39,42)^ have demonstrated that prediction formulas are more likely to overestimate in populations with lower salt intake and underestimate in those with higher intake. This was consistent with the trends presented in the subgroups of urinary Na intake levels.

The 24hUNa estimates by the Kawasaki formula tended to be overestimated, while those by the Tanaka and INTERSALT formulas were inclined to be underestimated, consistent with trends identified in Brazilian^(43)^, Iranian^(33)^ and Indian populations^(37)^. More accurate estimation results might be achieved by closely approximating the conditions under which the formula was established. Therefore, the estimates derived from the Kawasaki formula in fasting morning urine group (24·17 mmol/d) and in the group with salt intake group over 10 g/d (22·14 mmol/d) are more accurate compared with those obtained by the other two formulas.

Among the studies excluded from the sensitivity analysis, one focused on the elderly high-risk stroke population in rural area of Shaanxi province, and the other on elderly hypertensive patients in a rural area of the Ningxia Hui Autonomous Region. Both studies involved high-risk elderly populations with lower urine volumes and higher urine Na concentrations, resulting in significantly higher estimates compared with the measures. Although these two studies were not included in the final meta-analysis, there is a need to validate more accurate formulas in more diverse populations or in larger epidemiological studies within China.

There were two limitations in this study. First, the meta-analysis mainly evaluated the validity of the Kawasaki formula, the Tanaka formula and the INTERSALT formula, involving only 8369 participants from thirteen studies in fifteen provinces of China, which might limit its extrapolation. Second, correlation might not be the best measure to assess the validity in the current context of monitoring and evaluating public health programmes for population salt reduction^(44)^. A more comprehensive and in-depth evaluation of different formulas should be implemented in the future.

### Conclusion

The meta-analysis suggested that the Tanaka formula estimates exhibited less bias and higher correlation in the Chinese population. The time of collecting spot urine specimens and Na intake level of the sample population might be the main factors affecting the accuracy of the formula estimation.

## References

[1] Santos JA , Li KC , Huang L et al. (2020) Change in mean salt intake over time using 24-h urine versus overnight and spot urine samples: a systematic review and meta-analysis. Nutr J 19, 136. 33280602 10.1186/s12937-020-00651-8 PMC7720567

[2] World Health Organization (2023) SHAKE the salt habit: Technical package for salt reduction. https://iris.who.int/bitstream/handle/10665/250135/9789241511346-eng.pdf;sequence=1 (accessed March 2023).

[3] Jin A , Xie W & Wu Y (2020) Effect of salt reduction interventions in lowering blood pressure in Chinese populations: a systematic review and meta-analysis of randomised controlled trials. BMJ open 10, e032941. 10.1136/bmjopen-2019-032941 PMC7044858 32071177

[4] Hu X , Zhang XC , Ma JX et al. (2019) Application and evaluation of urine measurement at different times methods for estimating salt intake. Zhonghua yu fang yi xue za zhi (Chinese journal preventive medicine) 53, 530–533. 10.3760/cma.j.issn.0253-9624.2019.05.019 31091615

[5] Mann SJ & Gerber LM (2019) Addressing the problem of inaccuracy of measured 24-hour urine collections due to incomplete collection. J Clin Hypertens (Greenwich, Conn) 21, 1626–1634. 10.1111/jch.13696 PMC8030429 31631523

[6] Kamińska J , Dymicka-Piekarska V , Tomaszewska J et al. (2020) Diagnostic utility of protein to creatinine ratio (P/C ratio) in spot urine sample within routine clinical practice. Crit reviews clinical laboratory sciences 57, 345–364. 10.1080/10408363.2020.1723487 32058809

[7] Tan M , Wang C , Song J et al. (2022) Spot urinary sodium to monitor relative changes in population salt intake during the UK salt reduction programme. J Hypertens 40, 1406–1410. 35762479 10.1097/HJH.0000000000003166

[8] Huang L , Trieu K , Yoshimura S et al. (2020) Effect of dose and duration of reduction in dietary sodium on blood pressure levels: systematic review and meta-analysis of randomised trials. BMJ (Clinical Res Ed) 368, m315. 10.1136/bmj.m315 PMC7190039 32094151

[9] Kawasaki T , Itoh K , Uezono K et al. (1993) A simple method for estimating 24 h urinary sodium and potassium excretion from second morning voiding urine specimen in adults. Clin Exp Pharmacology Physiol 20, 7–14. 10.1111/j.1440-1681.1993.tb01496.x 8432042

[10] Tanaka T , Okamura T , Miura K et al. (2002) A simple method to estimate populational 24-h urinary sodium and potassium excretion using a casual urine specimen. J Hum Hypertens 16, 97–103. 11850766 10.1038/sj.jhh.1001307

[11] Brown IJ , Dyer AR , Chan Q et al. (2013) Estimating 24-hour urinary sodium excretion from casual urinary sodium concentrations in Western populations: the intersalt study. Am J Epidemiol 177, 1180–1192. 23673246 10.1093/aje/kwt066 PMC3664342

[12] Han W , Sun N , Chen Y et al. (2015) Validation of the spot urine in evaluating 24-hour sodium excretion in Chinese hypertension patients. Am J Hypertens 28, 1368–1375. 26009166 10.1093/ajh/hpv037

[13] Ma W , Yin X , Zhang R et al. (2017) Validation and assessment of three methods to estimate 24-h urinary sodium excretion from spot urine samples in high-risk elder patients of stroke from the rural areas of Shaanxi province. Int J Environ Res Public Health 14, 1211. 29019912 10.3390/ijerph14101211 PMC5664712

[14] Xu J , Du X , Bai Y et al. (2020) Assessment and validation of spot urine in estimating the 24-h urinary sodium, potassium, and sodium/potassium ratio in Chinese adults. J Hum Hypertens 34, 184–192. 31659230 10.1038/s41371-019-0274-z PMC7027967

[15] Du X , Fang L , Guo J et al. (2021) Validity of predictive equations for 24-h urinary sodium excretion at the population and individual levels among Chinese adults aged 18–69 years. Sci Rep 11, 22404. 34789756 10.1038/s41598-021-00513-1 PMC8599737

[16] Dong J , Yan Y , Fan H et al. (2019) Accuracy validation of 8 equations to estimate 24-hour sodium by spot urine in young adolescents. Am J Hypertens 32, 257–264. 30517605 10.1093/ajh/hpy178

[17] Zhao Y , Liu W , Liu S et al. (2020) Estimating 24-h urinary sodium excretion from casual spot urine specimen among hypertensive patients in Northwest China: the salt substitute and stroke study. Public Health Nutr 29, 1–7. 10.1017/S1368980019005019 PMC11574822 32345383

[18] Qian N , Jiang Y , Wang Y et al. (2021) Validity of five formulas in estimating 24-h urinary sodium via spot urine sampling in hypertensive patients living in Northeast China. J Hypertens 39, 1326–1332. 33323909 10.1097/HJH.0000000000002769 PMC8183493

[19] Sun Y , Wang H , Liang H et al. (2021) A method for estimating 24-hour urinary sodium excretion by casual urine specimen in Chinese hypertensive patients. Am J Hypertens 34, 718–728. 33491075 10.1093/ajh/hpab020

[20] Page MJ , McKenzie JE , Bossuyt PM et al. (2021) The PRISMA 2020 statement: an updated guideline for reporting systematic reviews. BMJ (Clinical Res Ed) 372, n71. 10.1136/bmj.n71 PMC8005924 33782057

[21] Sidik K & Jonkman JN (2003) On constructing confidence intervals for a standardized mean difference in meta-analysis. Commun Statistics - Simul Comput 32, 1191–1203.

[22] Rupinski MT & Dunlap WP (1996) Approximating Pearson product-moment correlations from Kendall’s Tau and Spearman’s Rho. Educ Psychol Meas 56, 419–429.

[23] Cheung MW & Vijayakumar R (2016) A guide to conducting a meta-analysis. Neuropsychology Rev 26, 121–128. 10.1007/s11065-016-9319-z 27209412

[24] Serra-Majem L , Frost Andersen L , Henríque-Sánchez P et al. (2009) Evaluating the quality of dietary intake validation studies. Br J Nutr 102, S3–S9. 20100366 10.1017/S0007114509993114

[25] Zhang Y , Peng Y , Li K et al. (2019) Assessing whether a spot urine specimen can predict 24-h urinary sodium excretion accurately: a validation study. J Hypertens 37, 99–108. 30063643 10.1097/HJH.0000000000001879

[26] Zhang X , Liao H , Ye R et al. (2021) Assessment and validation of three spot urine assay methods for the estimation of 24-hour urinary sodium excretion in Chinese Tibetan adults living in the mountains. J Clin Hypertens (Greenwich, Conn) 23, 1588–1598. 10.1111/jch.14312 PMC8678802 34196446

[27] Xu J , Zhang J , Liu M et al. (2020) Estimating 24-hour sodium excretion from spot urine samples in Chinese adults: can spot urine substitute 24-hour urine samples?. Nutrients 12, 798. 32197398 10.3390/nu12030798 PMC7146571

[28] Zhou L , Tian Y , Fu JJ et al. (2017) Validation of spot urine in predicting 24-h sodium excretion at the individual level. Am J Clin Nutr 105, 1291–1296. 28356277 10.3945/ajcn.116.147553

[29] Peng Y , Li W , Wang Y et al. (2016) Validation and assessment of three methods to estimate 24-h urinary sodium excretion from spot urine samples in Chinese adults. PloS one 11, e0149655. 26895296 10.1371/journal.pone.0149655 PMC4760739

[30] Wu B , Yang H , Ren X et al. (2022) A method for estimating 24 h urinary sodium and potassium excretion by spot urine specimen in stroke patients. Nutrients 14, 4105. 36235755 10.3390/nu14194105 PMC9573759

[31] Akoglu H (2018) User’s guide to correlation coefficients. Turk J Emergency Med 18, 91–93. 10.1016/j.tjem.2018.08.001 PMC6107969 30191186

[32] Peng YG , Feng JJ , Zhang Y et al. (2021) Cosinor-rhythmometry for 24-h urinary sodium, potassium, creatinine excretion in the Chinese adult population. Chin Med J 134, 539–545. 33410633 10.1097/CM9.0000000000001319 PMC7929596

[33] Hariri M , Ramezani AM , Shamshirgaran SM et al. (2023) Is a spot urine sample a good substitution to estimate 24-h urinary sodium excretion in a population ≥ 50 years old? A validation study. Eur J Nutr 62, 3277–3286. 37580619 10.1007/s00394-023-03217-6

[34] Polonia J , Lobo MF , Martins L et al. (2017) Estimation of populational 24-h urinary sodium and potassium excretion from spot urine samples: evaluation of four formulas in a large national representative population. J Hypertens 35, 477–486. 27898506 10.1097/HJH.0000000000001180

[35] Mente A , O’Donnell MJ , Dagenais G et al. (2014) Validation and comparison of three formulae to estimate sodium and potassium excretion from a single morning fasting urine compared to 24-h measures in 11 countries. J Hypertens 32, 1005–1014. 24569420 10.1097/HJH.0000000000000122

[36] Emeville E , Lassale C , Castetbon K et al. (2019) Estimating sodium intake from spot urine samples at population level: a validation and application study in French adults. Br J Nutr 122, 186–194. 31006386 10.1017/S0007114519000886

[37] Petersen KS , Johnson C , Mohan S et al. (2017) Estimating population salt intake in India using spot urine samples. J Hypertens 35, 2207–2213. 28697010 10.1097/HJH.0000000000001464

[38] Cogswell ME , Wang CY , Chen TC et al. (2013) Validity of predictive equations for 24-h urinary sodium excretion in adults aged 18–39 y. Am J Clin Nutr 98, 1502–1513. 24047921 10.3945/ajcn.113.059436 PMC3831536

[39] Huang L , Crino M , Wu JH et al. (2016) Mean population salt intake estimated from 24-h urine samples and spot urine samples: a systematic review and meta-analysis. Int J Epidemiol 45, 239–250. 26796216 10.1093/ije/dyv313

[40] Rhee MY , Kim JH , Shin SJ et al. (2017) Estimating 24-hour urine sodium from multiple spot urine samples. J Clin Hypertens (Greenwich, Conn) 19, 431–438. 10.1111/jch.12922 PMC8030767 27735123

[41] Kawamura M , Ohmoto A , Hashimoto T et al. (2012) Second morning urine method is superior to the casual urine method for estimating daily salt intake in patients with hypertension. Hypertens Res: Official Journal of the Japanese Society of Hypertension 35, 611–616. 10.1038/hr.2012.6 22297479

[42] Gong W , Ma Y , Zhang Z et al. (2022) Validation of 4 estimating methods to evaluate 24-h urinary sodium excretion: summer and winter seasons for college students in China. Nutrients 14, 2736. 35807918 10.3390/nu14132736 PMC9269089

[43] Mill JG , Rodrigues SL , Baldo MP et al. (2015) Validation study of the Tanaka and Kawasaki equations to estimate the daily sodium excretion by a spot urine sample. Rev brasileira epidemiologia = Braz J Epidemiol 18, 224–237. 10.1590/1980-5497201500060020 27008617

[44] Ji C , Sykes L , Paul C et al. (2012) Systematic review of studies comparing 24-hour and spot urine collections for estimating population salt intake. Rev panamericana salud publica = Pan Am J Public Health 32, 307–315. 10.1590/s1020-49892012001000010 23299293

